# A systematic review of dialectical behavior therapy mobile apps for content and usability

**DOI:** 10.1186/s40479-021-00167-5

**Published:** 2021-12-03

**Authors:** Chelsey R. Wilks, Kyrill Gurtovenko, Kevin Rebmann, James Williamson, Josh Lovell, Akash R. Wasil

**Affiliations:** 1 Department of Psychological Sciences, University of Missouri-St. Louis, 1 University Way, St. Louis, MO USA; 2 Department of Psychiatry and Behavioral Sciences, University of Washington, Washington, USA; 3 Seattle Children’s Hospital, Department of Psychiatry and Behavioral Medicine, Washington, USA; 4 Department of Psychology, Hofstra University, Hofstra, USA; 5 Department of Psychology, University of Pennsylvania, Pennsylvania, USA

**Keywords:** Dialectical behavior therapy, mHealth, Usability, Engagement

## Abstract

**Background:**

The gap between treatment need and treatment availability is particularly wide for individuals seeking Dialectical Behavior Therapy (DBT), and mobile apps based on DBT may be useful in increasing access to care and augmenting in-person DBT. This review examines DBT based apps, with a specific focus on content quality and usability.

**Methods:**

All apps referring to DBT were identified in Google Play and iOS app stores and were systematically reviewed for app content and quality. The Mobile App Rating Scale (MARS) was used to evaluate app usability and engagement.

**Results:**

A total of 21 free to download apps were identified. The majority of apps (71%) included a component of skills training, five apps included a diary card feature. Most (76.19%) apps were designed to function without help from a therapist. The average user “star” rating was 4.39 out of 5. The mean overall MARS score was 3.41, with a range of 2.15 to 4.59, and 71.43% were considered minimally ‘acceptable,’ as defined by a score of 3 or higher. The average star rating was correlated with the total MARS score (*r* = .51, *p* = .02). Estimates of app usage differed substantially between popular and unpopular apps, with the three most popular apps accounting for 89.3% of monthly active users.

**Conclusions:**

While the present study identified many usable and engaging apps in app stores designed based on DBT, there are limited apps for clinicians. DBT based mobile apps should be carefully developed and clinically evaluated.

## Background

Dialectical Behavior Therapy (DBT) is a third wave behavioral intervention designed for patients with complex and severe behavioral, emotional, and interpersonal dysfunction. DBT is an efficacious treatment for a wide range of clinical problems in adults, including suicidal behavior, nonsuicidal self-injury (NSSI), and borderline personality disorder [1–3]. It is also currently the leading treatment for NSSI and suicide attempts for adolescents [4–6]. Although DBT has been widely disseminated throughout the United States and globally (e.g. [7]), there remains a large proportion of the population who need DBT but cannot access the treatment [8]. Fully adherent DBT programs are scarce and do not match the high demand for this clinical service [9]. This gap between need and availability is particularly problematic for DBT given that many referred individuals present with acute life-threatening behaviors, and a lack of access to care can have significant negative long-term consequences for patients and their families.

One way of increasing availability of mental health treatment is through smartphone based mental health apps [10], and a number of consumer-facing mobile applications (i.e. mHealth) based on DBT have been developed to date. Unfortunately, there has been very little research on DBT mobile apps. Ilagan and colleagues (2020) recently published a systematic review and meta-analysis of smartphone applications designed to intervene on symptoms associated with borderline personality disorder (BPD) [11]—a behavioral disorder which is commonly treated using DBT. They found that only three studies included indicated the use of apps based on DBT. In one study, Rizvi and colleagues [12] found support for good acceptability and usability of a skills training focused app which was also associated with reductions in NSSI in a small clinical sample of 16 individuals receiving standard DBT. Another app based on DBT was developed and its usability and engagement was evaluated in a series of studies [13, 14]. While both of these apps were developed in collaboration with experts in DBT, neither apps are currently available for consumers to download in app stores. Importantly, while usability and engagement of mobile apps is an important first step in the mobile and computerized treatment development process, neither of these apps were tested in a randomized clinical trial (RCT), inhibiting the ability to establish their efficacy. Among apps that are currently available to consumers, it is unclear which contain essential elements integral to DBT, and even those apps which promote active ingredients of the treatment may vary widely in how usable or engaging they are.

Given the complex, modular, and comprehensive nature of the treatment, DBT mHealth apps can also be designed and implemented in a variety of ways. Some apps may be used as a standalone intervention, while others may be designed to technologically augment more traditional clinical care. Questions of what, when, and how technology is incorporated into mental health care are especially critical in the wake of Coronavirus 2019 (COVID-19). Safety recommendations and policies in response to COVID-19 pushed most outpatient psychotherapy for both adults and youth into a rapid transition to teletherapy [15, 16]. The recent shift to remote therapy has forced many DBT therapists to rapidly and flexibly adapt their implementation of DBT with little to no guidelines for how best to do so [16]. Leaning on technological supports such as mHealth apps may be one way in which clinicians and patients have adapted to utilizing DBT since the pandemic.

There are many essential elements of DBT that may be candidates for being delivered through or enhanced by mHealth apps. Research suggests that DBT skills training is one of the active ingredients of the treatment for both adults and adolescents [17–20], and app based DBT skills training and practice may be one of the most feasible and effective ways to port aspects of DBT to mHealth. Another essential ingredient of the treatment is the DBT diary card, which guides individual therapy, provides weekly progress monitoring and assessment of treatment targets, and facilitates agenda setting and suicide risk assessment and management. Completing diary cards through an app may be another effective way to integrate an essential element of DBT into the mobile user experience. There are also other critical elements of suicide focused treatments such as safety planning, access to crisis lines, and the ability to contact a therapist that can be incorporated into apps [21]. Unfortunately, at this stage it is unclear which essential elements of DBT are offered among available apps, and the extent to which these features are implemented with clinically useful depth and flexibility (e.g., is the diary card customizable? can it be shared with the therapist?).

Beyond unknowns about the range of DBT content represented across currently available DBT apps, the apps may also vary widely in their quality, engagement, and usability. Inconsistencies in engagement is one of the top challenges for using apps for mental health treatment, and aspects of the user experience are critical to the success of mHealth apps [10]. In this context, user experience refers to how engaging a mobile app is (i.e. how much a user’s interest is maintained) and how usable a mobile app is (i.e. how functional the features are). Although user engagement is important, it is not a proxy for an app’s efficacy, and many apps may misrepresent its “effectiveness” in treating a condition [22]; thus, it is important to disambiguate app efficacy from user engagement. Importantly, less than 2% of apps identified in app stores have research support [23], highlighting the importance of reviewing apps from mobile stores.

Based on our knowledge, we know of no research that has systematically reviewed or evaluated mobile DBT apps that are available in app stores. Guidance about which apps are most adequately designed to support technology assisted delivery of DBT is needed now more than ever, given the field’s current reliance on technologically assisted delivery of DBT. The current study systematically reviews and evaluates currently available DBT mobile apps with a focus on the range, characteristics of content, and user experience. In this context, user experience refers to the usability and engagement of a mobile app. We also examine which app characteristics are associated with user experience. Furthermore, we examine the extent to which these apps have disseminated to real-world users by obtaining estimates of active users. Finally, based on our findings, we discuss clinical considerations and recommendations for incorporating mobile apps into therapy.

## Methods

### App selection

Apps were initially identified in October 2020 through a systematic search of the United States (U.S.) iTunes and Google Play stores. Search terms included “dialectical behavior therapy,” “DBT,” “Marsha Linehan,” and “Linehan.” Apps were included if they: (a) were smart-phone based (b); used Android or iOS operating systems (c); were in the English language (d); had one or more of the aforementioned search terms in the app description; and (e) were available for download in the U.S. app store (iTunes or Google Play). Apps were excluded if they: (a) were not related to DBT (b); were not related to therapy or treatment (e.g. conference apps) (c) were not free; and (d) were not available for download or accessible. For the current study, apps were tested on a variety of devices, in part to simulate the wide range of devices that real world users of these apps may utilize to access them. We chose to focus on free apps to cover the range of apps that would be accessible to all users regardless of their financial resources to pay for such. iPhone apps were downloaded and tested using iPhones SE using iOS 14.4, iPhone 7 using iOS 14.4, and iPhone 11 using iOS 14.4. Android apps were downloaded and tested using a variety of devices, including a Galaxy Note 10+ on Android version 11 and One Plus 7 version 11.

### Data extraction

The following data about all apps were recorded: app name, platform (Android, iOS), current version number, cost, number of installs (Android only), and user ratings. The developer(s) of the apps were also categorized as being developed via a commercial entity, non-profit, clinician-led team, or unknown. In addition, as many apps are free to download but offer expanded content for a cost, we coded for whether the presence of a feature was included, not included, or whether it was available as a paid upgrade. We coded whether the app was primarily a DBT app (“DBT only”), or whether specific DBT components were included in addition to other psychotherapies (“DBT mixed”); this was determined by examining the app store description. Apps were also coded for their intended age range by examining the app store description and age ratings, as well as coding for whether there was any indication of whether the app was based on adolescent DBT (DBT-A) within app store descriptions or within app descriptions and content.

#### Content coding scheme

A DBT content coding scheme was developed by the first two authors for the purposes of coding for the presence of elements of DBT. DBT is a complex treatment which includes many protocols and treatment strategies, and we selected a handful of integral DBT treatment elements to code. Specific features that are known to enhance delivery of DBT and/or are integral to DBT were identified, such as: diary card, whether the diary card was customizable, skills training (including specific skills training modules, emotion regulation, distress tolerance, interpersonal effectiveness, mindfulness, and middle path), chain analysis, safety planning, access to a crisis line(s), access to a therapist, and the ability to send content and information to a therapist (such as a diary card).

DBT also contains a handful of essential stylistic and communication strategies -- that is, the style in which DBT content is delivered. For the current study we chose to code for the presence of validation and irreverence, given that these are two skills and stylistic elements that are essential to, and highly characteristic of DBT [24]. In the context of a mobile app, validation was coded as instances where the app was able to reflect back, reinforce, or express genuine understanding of a user’s potential emotion or experience. Irreverence was coded as instances of an app expressing messages that contained some sense of humor, sarcasm, lightheartedness or otherwise unexpected communication around DBT learning or practice tasks. We also coded for the inclusion of a feature for safety planning in each app. A safety plan consists of a prioritized list of coping skills and resources that can be quickly accessed and used to get through a suicidal crisis without acting on crisis urges [25]. Apps were further coded for whether the primary function of the app was to supplement face-to-face therapy or work as a standalone tool.

### Active user data

We acquired estimates of each app’s monthly active users (MAUs). Data were acquired from Mobile Action, a mobile app market research firm that provides estimates of app usage [26]. We obtained estimates for a one-month period from mid-April 2021 to mid-May 2021. In addition to reporting summary statistics (e.g., mean, SD, median) of the MAU data, we also report two additional metrics that can facilitate the interpretation of MAU data: the Market Share Index-3 (MSI-3) and the Number Needed to Reach-90 (NNR-90). The MSI-3 refers to the percentage of total MAUs that are accounted for by the 3 most popular DBT apps. The NNR-90 refers to the number of DBT apps that are needed to account for 90% of the total MAUs. Higher MSI values and lower NNR values indicate that the top apps are responsible for a greater proportion of MAUs [27].

### Mobile app user experience

All apps were rated by two independent reviewers using the 23-item Mobile App Rating Scale (MARS) to assess their usability and engagement. Each item was rated on a 5-point scale (1 = inadequate, 2 = poor, 3 = acceptable, 4 = good, and 5 = excellent) with descriptors provided for each anchor rating. Items in the MARS are grouped into 4 categories: engagement (5 items), functionality (4 items), aesthetics (3 items), and information quality (7 items). The MARS is scored with a mean for each of the categories and an overall total mean score. An overall mean score at or above a 3 indicates that the app is “acceptable” in terms of usability and engagement. The MARS has demonstrated good internal consistency (alpha = .90) and inter-rater reliability (ICC = .79) in previous research [28].

Before the app assessment, the four reviewers (CW, JW, JL, KM) discussed the use of the MARS in the context of apps for DBT treatment providers or consumers and each coder reviewed the training video on the MARS. Each app was double coded for iOS (CW, JW) and Android (JL, KM). We based the target audiences on the following user groups: patients and clinicians. After a consensus was achieved on the MARS, the reviewers independently rated the included apps. Each reviewer interacted with the identified app for several minutes, ensuring that all aspects of the functionality were tested and evaluated. When reviewers had questions or concerns related to the apps, these issues were discussed among authors, and a consensus was achieved.

### Statistical analyses

We conducted descriptive analyses to describe app content. For usability and engagement quality, scores were calculated for each MARS item, along with a total mean score. Interrater reliability of the MARS subscales and total quality score were calculated using the intraclass correlation coefficient (ICC) 2-way random-effects model of absolute agreement between single ratings. The mean value for each dimension of MARS was computed. Relations between MARS ratings and specific app characteristics were computed using Pearson’s *r* correlations. All statistical analyses were performed in R [29].

## Results

### Search results

**Fig. 1:**
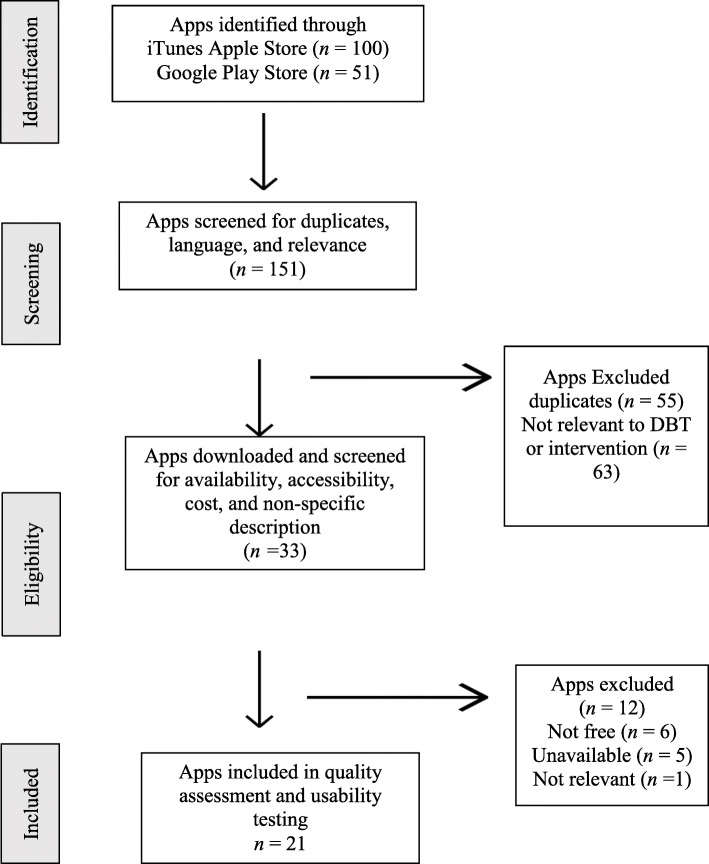
Systematic app selection

A total of 151 apps (iTunes Apple store, *n* = 100; Google Play Store, *n* = 51) were initially screened. In the screening stage, 55 apps were excluded because they were duplicates, not in the English language, or irrelevant (e.g., games, dialect/language apps). A further 63 were excluded because they were not relevant to DBT, and/or not intended for behavior modification/intervention. Of the 33 apps that were downloaded and tested for eligibility, 12 were excluded because they were no longer available or accessible for download, unrelated to DBT, or not free to download. The remaining 21 apps were included in the content and quality assessment and usability evaluation (Fig. 1).

### Descriptive characteristics of app content

#### Features included in DBT apps

Most apps (85.71%) included a privacy policy readily accessible within the app, while 33.33% of apps were password protected. Approximately half of the apps (47.62%) included notification as a feature. Related to app developers, the majority of apps (57.14%) were developed by a commercial entity, followed by a clinician-led team (19.05%), unknown developer (14.29%), and Non-profit (9.53%). Most apps (12/21) were determined to be DBT focused. Two apps specifically were advertised to be adolescent focused. The majority of apps (16/21) were considered stand alone as opposed to adjunctive. See Table 1.

**Table 1:** App content and features

App	Skills training	ER	DT	IE	MF	MP	Validation	Irreverence	Safety plan	Suicide hotline	Chain analysis	Diary card	Custom-ize diary card	Share diary card	Number of DBT features					Android rating	iOS rating	Android review	iOS	Monthly upgrade	Password	Privacy policy	Notifi-cations	MARS
																DBT only	Adolescent	App developer	Stand alone app			Totals	Review	Cost	Protected			Total
																							Totals					
Calm Harm - Manages Self Harm	Yes	No	Yes	No	Yes	No	Yes	No	Yes	Yes	No	No	NA	NA	3	Yes	No	Non Profit	Yes	4.3	4.4	1,780	511	0	No	Yes	No	3.8
CrisisBuddy	Yes	No	Yes	No	No	No	Yes	No	Yes	No	No	No	No	No	2	Yes	Yes	Clinician-Led	Yes	4.2	5	8	1	0	No	No	Yes	3.13
DBT Coach	UG	UG	UG	UG	Yes	UG	No	No	Yes	Yes	No	Yes	No	No	3	Yes	No	Commerical	Yes	4.5	4.7	1,404	742	$12.00	Yes	Yes	Yes	3.25
DBT Travel Guide	Yes	Yes	Yes	Yes	Yes	No	No	No	Yes	No	Yes	Yes	No	Yes	3	Yes	No	Clinician-Led	No	4.1	4.7	96	11	0	No	Yes	Yes	3.34
DBT Trivia and Quiz	Yes	Yes	Yes	Yes	Yes	No	No	No	No	No	No	No	No	NA	1	Yes	No	Commerical	Yes	4.1	4.3	14	234	0	No	Yes	No	2.4
DBT: The Dime Game	No	No	No	Yes	No	No	No	No	No	No	No	No	NA	NA	0	Yes	No	Unkown	Yes	3.1	4.8	17	4	0	No	Yes	No	3.95
Impulse DBT	No	NA	NA	NA	NA	NA	No	No	No	No	Yes	No	NA	NA	1	Yes	No	Unkown	No	4.1	3.9	105	14	0	No	No	Yes	2.86
Meela: Your Wellness Ally	Yes	UG	UG	NA	UG	No	Yes	No	Yes	Yes	No	No	NA	NA	3	No	No	Commerical	Yes	NA	4	NA	15	$7.00	Yes	Yes	No	3.52
Jones Mindful Living	UG	UG	UG	UG	UG	UG	No	No	No	No	No	No	NA	NA	1	No	No	Clinician-Led	Yes	--	NA	0	NA	$19.00	No	Yes	No	2.45
Mooditude - Elevate your mood	Yes	UG	UG	No	UG	No	Yes	No	No	No	No	No	NA	NA	2	No	No	Commerical	Yes	NA	4.7	NA	157	$15.00	Yes	Yes	Yes	4.37
Moodlinks	Yes	Yes	Yes	Yes	Yes	No	No	No	No	No	No	No	NA	NA	1	Yes	No	Commerical	No	4.6	5	29	27	0	No	Yes	Yes	3.42
Morpheus	No	No	No	No	No	No	No	No	Yes	Yes	No	No	No	No	1	Yes	No	Commerical	No	4.4	NA	5	NA	0	Yes	Yes	No	2.66
Psych Surveys	No	NA	NA	NA	NA	NA	No	No	No	No	No	Yes	Yes	Yes	1	No	No	Commerical	No	3.8	3.7	5	6	0	Yes	Yes	No	3.96
Resili	Yes	Yes	Yes	Yes	Yes	Yes	Yes	No	No	No	No	No	NA	NA	2	No	No	Commerical	Yes	4.7	5	15	1	0	Yes	Yes	Yes	3.37
Rise Up + Recover: An Eating Disorder Monitor	Yes	No	Yes	Yes	Yes	No	No	No	No	No	No	Yes	Yes	No	2	No	No	Non Profit	Yes	4.3	4.7	504	208	0	No	Yes	No	3.67
SafePlan	No	No	No	No	No	No	No	No	Yes	Yes	No	Yes	No	No	1	Yes	Yes	Unkown	Yes	--	--	--	0	0	No	Yes	Yes	2.59
Self-Heal	No	No	Yes	No	No	No	No	No	Yes	Yes	No	No	No	No	0	Yes	No	Clinician-Led	Yes	3.2	5	7	3	0	No	No	No	3.31
Skills, Stress Tolerance Game	Yes	No	Yes	No	No	No	No	No	No	No	No	No	NA	NA	1	Yes	No	Commerical	Yes	3.5	3.6	98	5	$5.00 (one-time)	No	Yes	No	2.15
Woebot - Your Self-Care Expert	Yes	Yes	Yes	No	Yes	No	Yes	Yes	Yes	Yes	No	No	NA	NA	4	No	No	Commerical	Yes	4.7	4.8	8,593	3,700	0	Yes	Yes	No	4.51
Wysa: Mental Health Support	Yes	UG	No	No	Yes	No	Yes	No	Yes	Yes	No	No	NA	NA	3	No	No	Commerical	Yes	4.7	4.8	74,350	3,500	$12.00	No	Yes	Yes	4.59
Youper	Yes	Yes	Yes	No	Yes	No	Yes	No	No	No	No	No	NA	NA	2	No	No	Commerical	Yes	NA	4.9	--	14,000	$3.80	No	Yes	Yes	4.35

Note. *ER* Emotion Regulation, *DT* Distress Tolerance, *IE* Interpersonal Effectiveness, *MF* Mindfulness, *MP* Middle Path, *MARS* Mobile App Rating Scale; Yes = content present, No = content absent, UG = content present and more is accessible with upgrade costs

#### Range of DBT components represented in DBT Mobile apps

The most common DBT content represented across apps was DBT skills, with 71.43% of apps including at least some type of DBT skills training content. Five apps (23.81%) included a diary card feature, and of those, two (Rise Up + Recover: An Eating Disorder Monitor and Psych Surveys) enabled users to customize their diary cards while two (DBT Travel Guide and Psych Surveys) enabled users to share their diary card with a therapist. Nine apps (47.92%) included a safety planning feature, which would include specific skills or outlines to manage current or future crises. In addition, 8 apps (38.10%) provided users with access to a suicide hotline. Six apps offered users an opportunity to pay for additional content, which included access to more features, therapy skills, or personalization (Table 1). Less than a quarter (22%) of apps offered a feature that allowed a therapist to contact or provide any feedback to the user, with one of these apps offering this feature as a premium paid option. Eight apps included features that were considered “validating,” which included cheerleading, encouragement, or acknowledgment of implementing the skills. One app (Woebot) was coded to have included some irreverent communication. Woebot is an app that includes a texting user interface which includes slight humor and unexpected responses to prompts. In general, the average number of features integral to DBT (e.g. diary card, access to suicide hotline, skills training, stylistic use of validation and irreverence, and chain analysis) was 2.29 (range 1–4), with Woebot including the most DBT features (see Table 1).

#### DBT only vs. DBT mixed

Most apps (57.14%) included only pure DBT components or were advertised as being based solely on DBT, while the remaining eight apps specifically advertised that DBT was integrated as an adjunct to other evidence-based interventions such as cognitive behavioral therapy. Other theoretical approaches mentioned in descriptions for some apps in the sample included Acceptance and Commitment Therapy (ACT), Emotion Focused Therapy (EFT), Prolonged Exposure (PE), Narrative Exposure Therapy (NET), Mentalization Based Therapy, Mindfulness Based Stress Reduction (MBSR), Functional Imagery Training, “eye movement therapy”, “mindfulness”, and art therapy. We further categorized the apps that included other evidence-based treatment components as “DBT mixed”, while apps that only advertised themselves as DBT apps were considered “DBT only.”

#### Standalone apps vs. apps designed to augment treatment as usual

Most apps (76.19%) were designed to function as standalone components while 5 apps (DBT Travel Guide, Impulse DBT, Moodlinks, Morpheus, & Psych Surveys) were designed to enhance or augment in-person or tele-DBT. Enhancement components included the ability to send crisis plans and diary cards to an individual therapist as well as track skills and dysfunctional behaviors. Stand-alone apps did not include these features, but included more teaching of specific therapeutic skills.

#### DBT vs. DBT-A and age ratings

Age ratings derived from the Apple App Store indicated that 6% of apps were rated appropriate for 17 +, 72% were rated for 12 +, and 22% were rated as 4 +. Content ratings derived from the Google Play app store indicated that 100% of the apps in the sample were rated appropriate for “everyone”. In app age guidelines were available for only 2 apps in the sample: “meant for young adults” (CrisisBuddy) and “designed to be used by those aged 16 years and older” (SafePlan). Only 1 (4.76%) app was explicitly designed for adolescents, and the rest of the included apps did not indicate an age range for which they were designed. Three apps (14.29%) contained middle path skills content, which was originally designed specifically for DBT-A [30].

### Monthly active user data

**Fig. 2:**
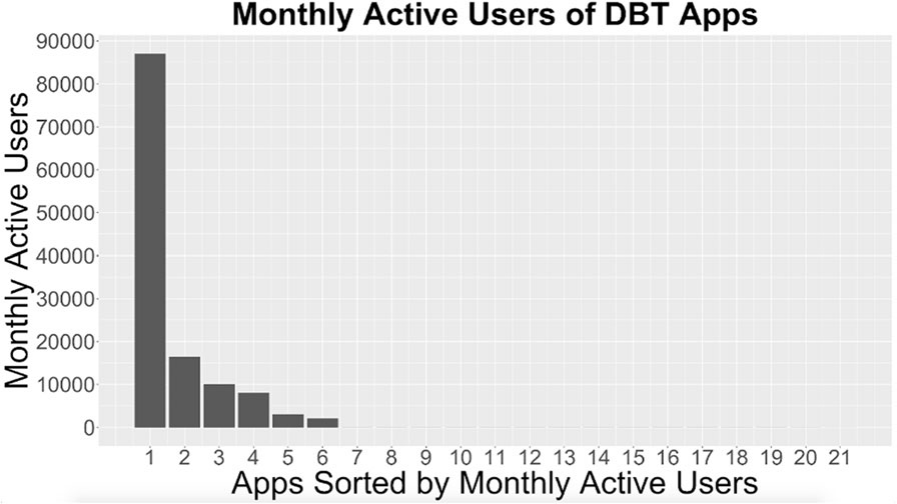
Monthly active users of DBT Apps

Fig. 2 shows the monthly active users for each app (Mean = 6050, SD = 19,039, Median = 36). The MSI-3 was 89.3%, indicating that the top three apps (Wysa, Youper, and Woebot) were responsible for 89.3% of MAUs. The NNR-90 was 4, indicating that the top four apps (the three aforementioned apps and Calm Harm) accounted for over 90% of MAUs.

### Mobile app user experience

The interrater reliability for usability ratings was in the acceptable range for each subscale (.61–.85), with the highest interrater reliability of .85 (engagement) and the lowest of .61 (aesthetics). The average user star rating was 4.39 out of 5 (total range: 3.1–5.0; iOS = 4.56, range 3.6–5.0; Android = 4.14, range 3.1–5.0) on an average of 2702.20 reviews (iOS =1217; Android = 4480). The mean overall MARS score was 3.41, with a range of 2.15 to 4.59, and 71.43% of apps had a minimum acceptability score of 3.0. In general, the*“*Wysa: Mental Health Support” app had the highest MARS overall score (4.59) followed by “Woebot” (4.51), and*“*Youper*”* (4.35). We examined how MARS scores differed in relation to the presence of specific DBT features.

#### Preliminary comparisons of Mobile apps user experience

The MARS total and subscale scores were all significantly correlated, indicating that app quality was consistent across the areas assessed (e.g., apps scoring high on engagement also tended to score high on function, aesthetics, and information). The overall MARS score was correlated with the user app rating (r = .51, *p* = .02), average number of reviews (r = .53, *p* = .01), and number of DBT features included within the app (r = .61, *p* = .003) indicating a high concordance with the MARS total score and our ratings (Table 2).

**Table 2:** DBT app MARS correlations with user rating, reviews, and features

Characteristics	MARS					Ave Star Rating	Ave number of reviews	Number of Features
	Total	Engagement	Function	Aesthetics	Information			
**MARS**								
Total	1.00							
Engagement	.91**	1.00						
Function	.87**	.66**	1.00					
Aesthetics	.87**	.72**	.69**	1.00				
Information	.78**	.69**	.59*	.53*	1.00			
Ave Star Rating	.51*	.58*	.18	.59*	.26	1.00		
Ave number of reviews	.53*	.60*	.38	.49*	.28	.41	1.00	
Number of Features	.61*	.74**	.37	.59*	.33	.49*	.52*	1.00
Mean (SD)	3.41 (0.72)	3.05 (0.92)	3.82 (0.81)	3.48 (0.78)	3.24 (0.79)	4.39 (0.39)	2702.21 (7762.82)	2.29 (1.27)

MARS = Mobile App Rating Scale; Ave = Average; SD = standard deviation ** *p* < .001; * *p* < .01

In order to quantify apps based on usability scores and specific features, apps were categorized in the following manner: DBT-only apps vs DBT mixed apps, Standalone app vs Non standalone app, and completely free app vs upgradable app. DBT-apps, on average, had slightly lower MARS scores than non-DBT apps (M = 3.16, SD = .66; M = 3.82, SD = .66) and more features (M = 1.76, SD = .91; M = 2.63, SD = 1.66). Standalone apps had slightly lower MARS scores than non-standalone apps (M = 3.25, SD = .51; M = 3.46, SD = .78), and less features (M = 1.20, SD = 1.37; M = 2.37, SD = .54). Finally, completely free apps had slightly lower MARS scores compared to upgradable apps (M = 3.71, SD = .93; M = 3.29, SD = .62), and less features (M = 1.87, SD = 1.06; M = 3.33, SD = 1.21). DBT-focused and free apps scored lowest in engagement (*M* = 2.76 *SD* = .69; *M* = 2.65, *SD* = .88). Free apps were relatively functional (*M* = 4.04, *SD* = .85), and standalone apps scored relatively poorly in the information subscale (*M* = 2.90, *SD* = .95).

## Discussion

We examined the content, features, usability, and engagement of mHealth apps based on DBT. There were several notable findings. First, there are several apps available based on DBT, yet they varied greatly in scope, features, and function. Most apps included aspects of skills training, while only a fraction included other crucial aspects to DBT delivery such as a diary card or chain analysis. Approximately half of the apps were solely based on DBT, while the others integrated DBT components to complement other therapy skills, and most apps were designed to be used without a therapist. A few highly popular apps attracted tens of thousands of users, with most apps having fewer than 50 monthly active users. The majority of the apps scored in the usable range. User ratings, number of features, and number of reviews were positively correlated with MARS scores, indicating overall agreement in app quality. Finally, the quality and feature selection available within DBT apps differed based on both the cost of the app and whether the apps were solely focused on DBT or merely included some components of DBT adjunctively with other psychotherapy content.

Our systematic review of mobile apps based on DBT revealed 21 apps that were downloadable, free, and functional. Though we identified 33 apps based on DBT, we were unable to code them all because many were unable to be downloaded or were not free. In an effort to streamline the delivery of DBT, a certification process was adapted, which involves testing and formal adherence ratings. There does not exist a similar process for the development and distribution of mobile apps based on DBT. As a result, the apps that we identified varied greatly in quality, content, and function. Integral to the delivery of DBT is daily tracking of behaviors via a diary card as well as skills use. DBT skills training was the most commonly represented component of DBT across the apps reviewed. This finding was not surprising, given that the skills mode of DBT is the most portable, adaptable, and widely disseminated part of the treatment [31, 32]. Moreover, DBT skills are most easily translatable into a mobile format. DBT skills are discrete, modular, follow a protocol, and some even include a flow chart [33], which make the skills particularly amenable to computerization. Stylistic features of DBT such as irreverence and other essential treatment protocols like chain analysis, were less frequently integrated, likely due to the complexity of translating such highly idiographic and interpersonally interactive elements. Nonetheless, only 23% of apps included a diary card, and of those, only two offered the ability to customize it and share it directly with a therapist through the app. Apps designed to track behavior, thoughts, and emotions are common [34], which is why an app based on DBT that does not include this feature is surprising. Slightly less than half of the apps included features for crisis management and more than a third of apps provided users access to suicide hotlines. These features are particularly important as DBT is considered one of the most effective interventions of choice for those at high risk for suicide [1]. Having access to crisis planning and hotlines may be particularly important with apps that are designed to be delivered in the absence of a trained clinician.

We found that there were large differences in usage between the apps. The three most popular apps accounted for 89% of the total monthly active users. While some apps had thousands of users, half of the apps had 36 or fewer active users. This immense divide between the popular apps and unpopular apps is striking. One plausible explanation is that the highly popular apps (Wysa, Youper, Woebot, and Calm Harm) are transdiagnostic and offer a variety of content, whereas other apps (e.g., DBT Coach, DBT Travel Guide, DBT Trivia and Quiz) are narrower in their focus. Apps like Wysa and Youper may attract users with a broader variety of concerns than apps that exclusively or primarily offer DBT. Another plausible explanation is that users generally tend to gravitate toward a small number of highly engaging and well-advertised apps. Our findings are not unique to DBT apps: previous research has found similar distributions of active users in apps for depression and anxiety [35], eating disorders [36], and other health conditions [27]. Thus, the large differences between popular and unpopular apps may not be as surprising as it may appear at first glance. Future research will be needed to understand specifically why and how the popular mHealth apps successfully attract and retain users.

Our review identified several differences between apps that were advertised as being solely based on DBT vs. apps that included some aspects of DBT to complement a suite of other therapeutic techniques. Apps that were advertised to be solely focused on DBT seemed to be designed with a specific function in mind. For example, the app “DBT: The Dime Game,” is designed to support practice and implementation of one specific DBT skill while “Impulse DBT” is designed to walk users through a chain analysis--a crucial component in individual DBT. These apps suffered in the amount of breadth of “DBT specific” features that they contained, yet were clearly designed to support more in depth features around essential DBT tasks. Apps like Youper and Wysa integrated specific DBT skills within a toolbox of evidence-based interventions designed to improve a broad class of symptoms. While the apps that integrated components of DBT tended to be highly usable and engaging, pulling from a mix of treatments for an app may lead to a confusing or disorienting user experience. For example, someone searching for DBT based support may find it difficult to understand how much DBT is contained in an app that advertises other treatments, or whether any particular skill or content in the app is DBT based or not. On the other hand, apps that draw on a range of evidence-based approaches are philosophically and theoretically consistent with DBT, in the sense that DBT utilizes whatever is proven to work to achieve clinical progress. Our usability analysis also showed that mixed-DBT apps showed just as high, if not higher, usability ratings, suggesting that adding other evidence-based treatments to a well-designed DBT app may not compromise the user experience.

Apps that included integration with a therapist (such as sending a diary card to a therapist) were coded as “adjunctive;” most apps were designed specifically for skills practice in the absence of therapy while less than a quarter were designed to augment in person treatment. DBT along with other evidence-based treatments were originally designed to be delivered in person; however, the COVID-19 pandemic has likely permanently shifted how outpatient therapy is delivered. While many clinicians report difficulties delivering treatment via telehealth, the convenience for both patients and providers indicate that at least some proportion of telehealth delivered mental health care is here to stay [37, 38]. As a result, effective technologies and mobile applications that can support teletherapy are needed now more than ever.

Our results revealed an overall lack of attention to age and developmental factors with regard to DBT apps in the sample. Only two apps were explicitly designed to be appropriate for younger people, while most apps were generally described as being appropriate for “everyone”. Adolescents are particularly amenable to mobile mental health, with 95% of adolescents reporting that they own a smartphone [39], and approximately 64% of adolescents reported using apps to manage mental health symptoms [40]. Practitioners and researchers of DBT for adolescents and youth have long advocated for the importance of developmentally sensitive modifications to DBT when applied for this population [30, 41]. Although some apps in the current sample may be effective for adult users, they may be less appropriate for youth for a variety of reasons such as containing content written at too complex of a reading level, not being engaging or “youth friendly” enough, or not addressing or acknowledging the importance of the family context of youth. Future work in this area should remain sensitive to how DBT based apps can be developed to be efficacious and well adapted to the full age range of DBT mHealth users. Otherwise, DBT mHealth app developers may consider developing different versions of effective DBT apps that are specifically tailored to be developmentally appropriate (e.g., DBT adult apps, DBT adolescent apps, and DBT apps for children).

The significant positive associations between MARS scores and other indices of app quality suggests that both DBT clinicians and patients can use app ratings and reviews as a proxy for app quality; however, it should be noted that the correlations were not particularly strong with expert ratings. Moreover, the large positive correlation between MARS scores with user star ratings, number of ratings, and features indicates that apps that are downloaded frequently and have several features tend to win over users and raters. Engagement in mHealth is a critical, but often overlooked aspect in app development. In general, most users of mobile mental health apps tend to stop using an app after 10 uses [42], and given mHealth use is associated with clinical outcomes [13], how the content is designed and displayed is as important as the content itself. To note, the majority of the apps were developed by a commercial entity and some of the highest rated apps included a “pay for more” feature, indicating a business model that could sustain the app development, updating, and maintenance. The median cost of developing a smartphone app is $171,000, and yearly maintenance costs is estimated to be around 20% of development costs [43]. As such, it may be too expensive and infeasible for researchers to develop and maintain their own apps. Additionally, it appears to be the case that only a small number of apps has attracted and retained active users. However, the consequence of the costly app development is the lack of research supported apps on app stores, relegating usable and engaging apps to be developed by business. To note, neither the Behavioral Tech developed DBT coach (e.g. 11) nor Pocket Skills (e.g. 12) were identified in this app review because they are not available to download, highlighting a “lab to marketplace” gap for apps developed in the context of research.

### Limitations

While this is the first review that has systematically reviewed mobile apps based on DBT for content quality and user experience, there are several limitations that need to be addressed. One of the most glaring omissions in this review is the lack of concurrent systematic review to evaluate the apps’ clinical efficacy, as such a review would be outside the scope of this study. As a result, we are only able to comment on the apps usability and engagement, rather than potential efficacy. In addition, we did not thoroughly review mobile apps that required payment to download, limiting the breadth of our review. The inclusion of paid apps may have shifted the pattern of findings in the current study, that is, apps that required payment may have more features and may have been more engaging. However, clinicians report that the move to teletherapy has disproportionately affected low-income patients [44], and non-free mobile apps exclude these patients from the marketplace. Another limitation is that our app selection process only included a systematic search of iOS and Android app stores, and no systematic literature search was performed. We relied primarily on descriptive statistics with this small sample size, which limited our ability to draw statistical inferences from the observed patterns in the current sample. Thus, trends and patterns described in the current study should be interpreted cautiously. Although our content coding included some essential DBT components such as skills training and the diary card, there are many features of DBT that we did not code or discuss in the context of mHealth apps. Future studies on DBT mHealth apps should aim to expand coding and analyses to characterize a broader range of DBT features in order to further assess how well this complex treatment can be translated into apps and technology. Finally, the coders in this study were researchers rather than individuals with lived experience with DBT. Future studies should analyze DBT apps through the lens of patients and individuals with lived DBT experience as well as consumers of mental health apps.

## Conclusions and recommendations

Our DBT app search revealed a relatively high number of apps for download when seeking mHealth DBT support. Probably one of the most glaring omissions related to DBT mobile apps is the lack of research related to the efficacy of these apps as a standalone intervention or component to augment treatment. Relatedly, there does not appear to be any guidelines or quality control related to how mHealth apps are published on app stores; however, such guidelines have yet to be developed. At this time there may be some portions of DBT that can be technologically delivered, supported, or enhanced through DBT based apps, and the state of this technology is far from replacing the need for well-trained DBT therapists to ensure quality care; however, as mentioned, there is no indication whether these apps are efficacious. We recommend that both clinicians and patients use app ratings to make an informed decision about which apps might be best suited to their needs. As it relates to mobile app developers and designers, a closer partnership between DBT experts and app developers is needed in order to design an app that is maximally useful and clinically relevant. Finally, the current review poses many further questions that warrant future research. DBT is a relatively complex psychotherapy which relies on both discrete treatment components (e.g., didactic skills training) as well as more fluid active ingredients such as stylistic strategies. Research is needed to determine when and for whom integration of technology into DBT is most useful or needed, and which elements of DBT delivered through apps enhance treatment outcomes.

## Data Availability

The datasets during and/or analyzed during the current study available from the corresponding author on reasonable request.
